# Accuracy assessment of global land cover datasets for monitoring forest landscape restoration targets in Malawi

**DOI:** 10.1007/s10661-026-15733-z

**Published:** 2026-07-27

**Authors:** Tonthoza Uganja, Eefke Mollee, James Gibbons

**Affiliations:** School of Environmental and Natural Sciences, Bangor University, Bangor, Gwynedd UK

**Keywords:** Accuracy assessment, Forest restoration monitoring, Mosaic landscapes

## Abstract

Malawi contributes to the global restoration movement through its commitments under the UN Decade on Ecosystem Restoration and the Global Biodiversity Framework. With deforestation rates among the highest in sub-Saharan African countries, Malawi has pledged to restore 4.5 million hectares by 2030, creating an urgent need for reliable monitoring frameworks. Global 10-m-resolution land use and land cover (LULC) datasets derived from remote sensing are increasingly used to track progress in forest restoration. However, their reliability in heterogeneous, smallholder-dominated landscapes remains uncertain. This study evaluates the accuracy of three widely used global products, Esri Land Cover, ESA WorldCover, and Dynamic World, against > 5000 points from Malawi’s National Forest Inventory (NFI) data. All reported accuracy metrics are design-unadjusted. Results show that Esri achieved the highest overall accuracy (65.6%, 95% CI 64.2 to 67.0), followed by ESA WorldCover (61.9%, CI 60.5 to 63.3), while Dynamic World performed considerably lower (46.9%, CI 45.6 to 48.4). Class-specific performance revealed that tree cover was consistently well detected (user’s accuracy > 88%), whereas cropland and built-up areas were systematically underrepresented, and rangeland was overpredicted. As global calls for ecosystem integrity monitoring grow stronger, our findings show that global datasets, although valuable for broad and temporal coverage, cannot independently support national-scale FLR monitoring in mosaic landscapes such as Malawi. We recommend integrated monitoring frameworks that combine the global consistency of Earth observation with the credibility of national inventories and the contextual relevance of community-based data. Such approaches are essential to capturing the complexities of land use mosaics and supporting more accurate, legitimate, and policy-relevant restoration planning.

## Introduction

Global forest loss and degradation have been a primary concern in recent decades, with significant implications for biodiversity, ecosystem services, and climate change mitigation (Fagan et al., 2020). Forests are not homogeneous expanses but often have varied land use patterns, including untouched, degraded, and regenerating wooded areas (Brockerhoff et al., 2017). These diverse landscapes are called mosaic landscapes, characterised by their patchwork of varying forest qualities and land uses (Grantham et al., 2020). An expanding worldwide restoration initiative has gained momentum to address this issue, focusing on increasing tree and forest cover across diverse terrains through forest and landscape restoration (FLR) efforts (Erbaugh et al., 2020).

FLR is a comprehensive approach aimed at regaining ecological integrity and enhancing human well-being in deforested and degraded landscapes (Chazdon & Uriarte, 2016; Chazdon et al., 2020). Malawi exemplifies the complexity of restoration in mosaic landscapes, where forests, croplands, and agroforestry systems coexist and interact. The country’s land system is dominated by the Miombo woodland ecology, a socio-ecological matrix that supports both ecological processes and smallholder livelihoods (Mtambo & Missanjo, 2014; Shamaoma et al., 2022). However, this ecological mosaic is under increasing pressure from agricultural expansion, unsustainable fuelwood extraction, and overgrazing (Bone et al., 2017; Hermans et al., 2020). The result is a fragmented landscape in which natural and managed ecosystems are tightly interwoven, making it challenging to delineate ‘forest’ from ‘non-forest’ areas using global datasets. Recognising these challenges, Malawi, like many other African countries, has developed and adopted a national FLR strategy that outlines critical interventions, such as reforestation, agroforestry, and the establishment of community-managed forest areas to restore degraded landscapes, improve forest cover, and enhance biodiversity conservation (Kpienbaareh et al., 2022).

Despite these efforts, Malawi continues to experience high rates of deforestation, losing approximately 33,000 hectares of forest annually (Skole et al., 2021). This underscores the need for effective monitoring and evaluation tools to track progress towards FLR targets and to inform adaptive management. Malawi’s National Forest Landscape Restoration Strategy (Government of Malawi, 2017) operationalises forest policy objectives and aligns them with the global goals of climate change mitigation, biodiversity conservation, and sustainable development, and reflects Malawi’s commitment to restore 4.5 million hectares of degraded landscapes by 2030 under the African Forest Landscape Restoration Initiative (AFR100) and the Bonn Challenge (Djenontin et al., 2022; Messinger & Winterbottom, 2016). Implementation is coordinated through district-level plans and a participatory National Forest Landscape Restoration Assessment that identifies priority areas and interventions, including agroforestry, soil and water conservation, and forest management (Djenontin et al., 2021). Large-scale tree planting, catchment conservation, and other FLR interventions are now being implemented country-wide (Hermans et al., 2020), but monitoring must be progressive and continuous if targets are to be met, with rapid feedback to flag both negative trends and scalable successes.

Assessing the success of FLR initiatives in Malawi relies on accurately monitoring changes in land use and land cover (LULC) over time (Höhl et al., 2020). Global LULC datasets offer a potentially valuable resource, providing spatially comprehensive and temporally consistent data on forest cover dynamics (Hansen et al., 2021; Potapov et al., 2022). These datasets, derived from satellite imagery and remote sensing technologies, enable the assessment of forest cover gain or loss at various scales, from local to global (Almeida et al., 2019).

Several global LULC datasets have been instrumental in monitoring forest changes (Venter et al., 2022). These datasets, derived from remote sensing data, offer high-resolution imagery, frequent updates, and broad spatial coverage, making them suitable for tracking FLR progress in Malawi (Kadzuwa, 2023). However, the effectiveness of these global LULC datasets in monitoring forest landscape restoration targets in Malawi and comparable countries has not been thoroughly evaluated (Kadzuwa, 2023). Comparable validation of global land cover products against reference data has been reported for agriculture elsewhere in sub-Saharan Africa (Kerner et al., 2024), but no equivalent assessment against National Forest Inventory data has been carried out for Malawi. There are challenges related to data resolution, accuracy, and the ability to detect small-scale changes in heterogeneous landscapes like the Miombo woodlands (Shamaoma et al., 2022). Furthermore, the applicability of global datasets to local contexts requires validation to ensure that they can accurately reflect the ground realities of forest cover dynamics in Malawi (Hao et al., 2023).

This study provides a systematic assessment of the accuracy of multiple global 10-m-resolution LULC products against Malawi’s National Forest Inventory reference data. By evaluating their ability to detect forest and tree cover, as well as other classes relevant to FLR, the research provides critical insights into the strengths and limitations of using global datasets for national-scale FLR monitoring. The findings contribute to the development of robust monitoring frameworks to support the implementation and tracking of FLR initiatives in Malawi and similar contexts. Given the high rates of deforestation and Malawi’s ambitious 4.5-million-hectare restoration target, there is an urgent need for reliable and accurate monitoring tools (Kpienbaareh et al., 2022). This study addresses that need by evaluating the Esri, ESA WorldCover and Dynamic World global LULC datasets, contributing to the effective monitoring and management of forest restoration efforts in Malawi.

## Materials and methods

### Study area description

**Fig. 1:**
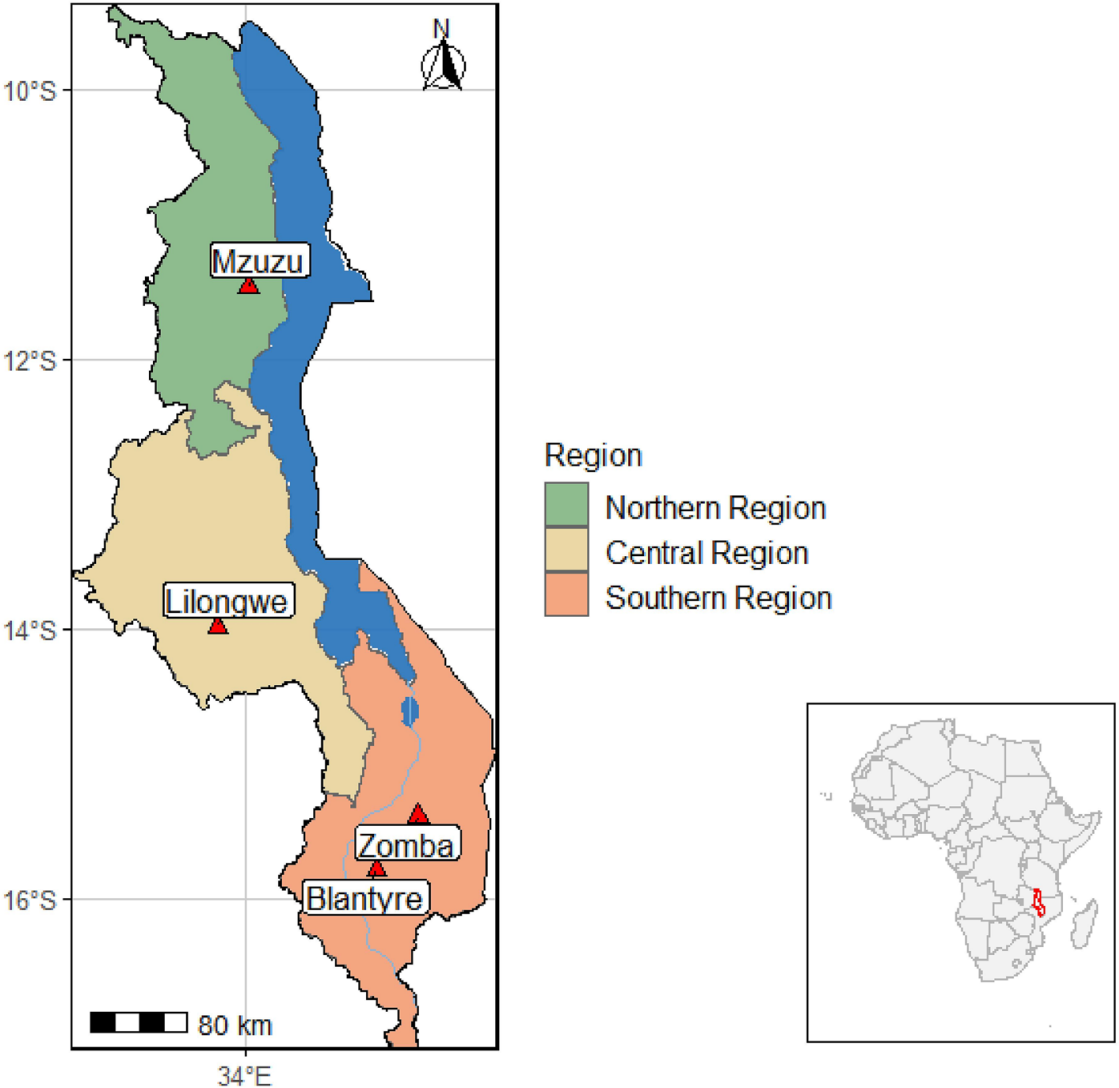
Study map of Malawi showing the three administrative regions (Northern, Central, Southern) used in this study. Major cities (Lilongwe, Blantyre, Mzuzu, Zomba) are marked with red triangles. Gridlines represent degrees of latitude and longitude, and the inset map highlights Malawi's location on the African continent

Situated between latitudes 9° and 18°S and longitudes 32° and 36°E, Malawi (Fig. 1) is located in southeastern Africa. With an area of approximately 11.8 million hectares and a population estimated at 22 million (UNFPA, 2025; World Bank, 2026), it shares borders with Mozambique on the east, south, and west; Tanzania to the northeast; and Zambia to the northwest (Caruso & Sosa, 2022). Malawi experiences distinct dry seasons alternating with wet seasons throughout the year. The predominant ecosystem across most of Malawi is Miombo woodland, primarily consisting of the genera *Brachystegia*, *Isoberlinia*, and *Julbernardia* (Chinangwa et al., 2017). This places Malawi within the Zambezian Miombo eco-region of sub-Saharan Africa, which contains one of the largest continuous expanses of plant life (Coutts et al., 2019). These woodlands are embedded in heterogeneous mosaic landscapes, where cropland, settlements, and tree cover coexist at fine spatial scales, making accurate land cover monitoring particularly challenging (Kerner et al., 2024; Xu et al., 2024). Malawi is among the most densely populated countries in sub-Saharan Africa, with a national average of approximately 200 people per km^2^, rising to more than 300 people per km^2^ in parts of the Southern Region (Caruso & Sosa, 2022). Agriculture is dominated by smallholder cultivation, with the average household landholding of around one hectare, typically subdivided into two or three discontinuous plots (Chinangwa et al., 2017; Hermans et al., 2020). This combination of high rural population pressure and very small, fragmented farm units produces a fine-grained patchwork of cultivation, fallow, scattered homesteads, and tree cover that frequently falls below the effective discrimination of 10-m global mapping products (Kerner et al., 2024; Xu et al., 2024).

### LULC data products and reference data

#### Data products

This study evaluates three global 10-m land use/land cover datasets: Esri Land Cover, ESA WorldCover, and Dynamic World, each produced using Sentinel-1/2 imagery and machine-learning classification pipelines (Brown et al., 2022; Karra et al., 2021; Xu et al., 2024; Zanaga et al., 2021). These datasets are widely used in restoration monitoring due to their global coverage and temporal frequency; however, their reliability in smallholder mosaic landscapes, such as those in Malawi, remains uncertain.

ESRI’s 10-m resolution annual land use/land cover (LULC) dataset leverages state-of-the-art deep learning techniques (Karra et al., 2021). The land cover and land use classification algorithm was trained on an extensive dataset of over 5 billion human-labelled pixels, based on 24,000 training sites distributed across all major biomes globally. Sentinel-2, a high-resolution multispectral satellite mission, has been extensively employed in land monitoring and has been shown to significantly enhance the spatial and spectral capabilities for land cover mapping compared to previous products, with a 10-m per pixel resolution (Xu et al., 2024).

ESA WorldCover v100 (2020) was produced by ESA using Sentinel-1 and Sentinel-2 imagery. WorldCover provides 11 global land cover classes at 10-m resolution, designed for policy monitoring and global assessments (Zanaga et al., 2021).

Dynamic World v1 (2020) (DW) was generated in near real time from Sentinel-2 imagery using Google Earth Engine. Dynamic World provides per-pixel class probabilities for nine classes, aggregated here into the four FLR-relevant categories (Brown et al., 2022).

#### Reference data

Malawi’s National Forest Inventory (NFI) was established to monitor forest resources, assess deforestation and degradation, and facilitate the attainment of forest landscape restoration goals (Kadzuwa, 2023). The data points for NFI were created using national LULC datasets sourced from several organisations, ensuring comprehensive and detailed coverage. The selection process for these points involved a thorough review of documentation, existing analyses, and GIS-based evaluations (MCHF, 2021). Malawi’s LULC classification system comprises six primary classes: forestland, cropland, grassland, wetlands, settlements, and other lands, each with specific definitions and thresholds aligned with national and international standards (IUCN, 2017). Monitoring activities entail regular assessments using field surveys, remote sensing technologies, and GIS analysis to validate and update the data points (IUCN, 2017).

Forestland is defined as land with woody vegetation that meets specific thresholds, such as minimum mapping area, crown closure at maturity, and the height and width of linear features, based on the LULC standards developed in collaboration with the Government of Malawi. Cropland includes various types such as agricultural land (Tea, Rain-fed herbaceous, Rice, etc.), with specific classifications like cultivated ‘Dambos’ (seasonally waterlogged areas found in the headwater zones of drainage systems or alongside streams (Turner, 1986) and agriculture in forest areas. Grassland encompasses categories like Herbaceous closed and Savanna, representing different types of grassy areas within the landscape. Wetlands comprise permanent marshes, Dambos (temporarily flooded areas), and other natural water bodies, which are distinguished by their characteristics. Settlements include built-up urban and non-urban areas, reflecting the presence of human habitation and infrastructure within the landscape. Other land consists of categories such as bare rock, riverbed, or beach, representing land types not classified under the primary categories like forest, cropland, settlements, grassland, and other land (MCHF, 2021).

**Fig. 2:**
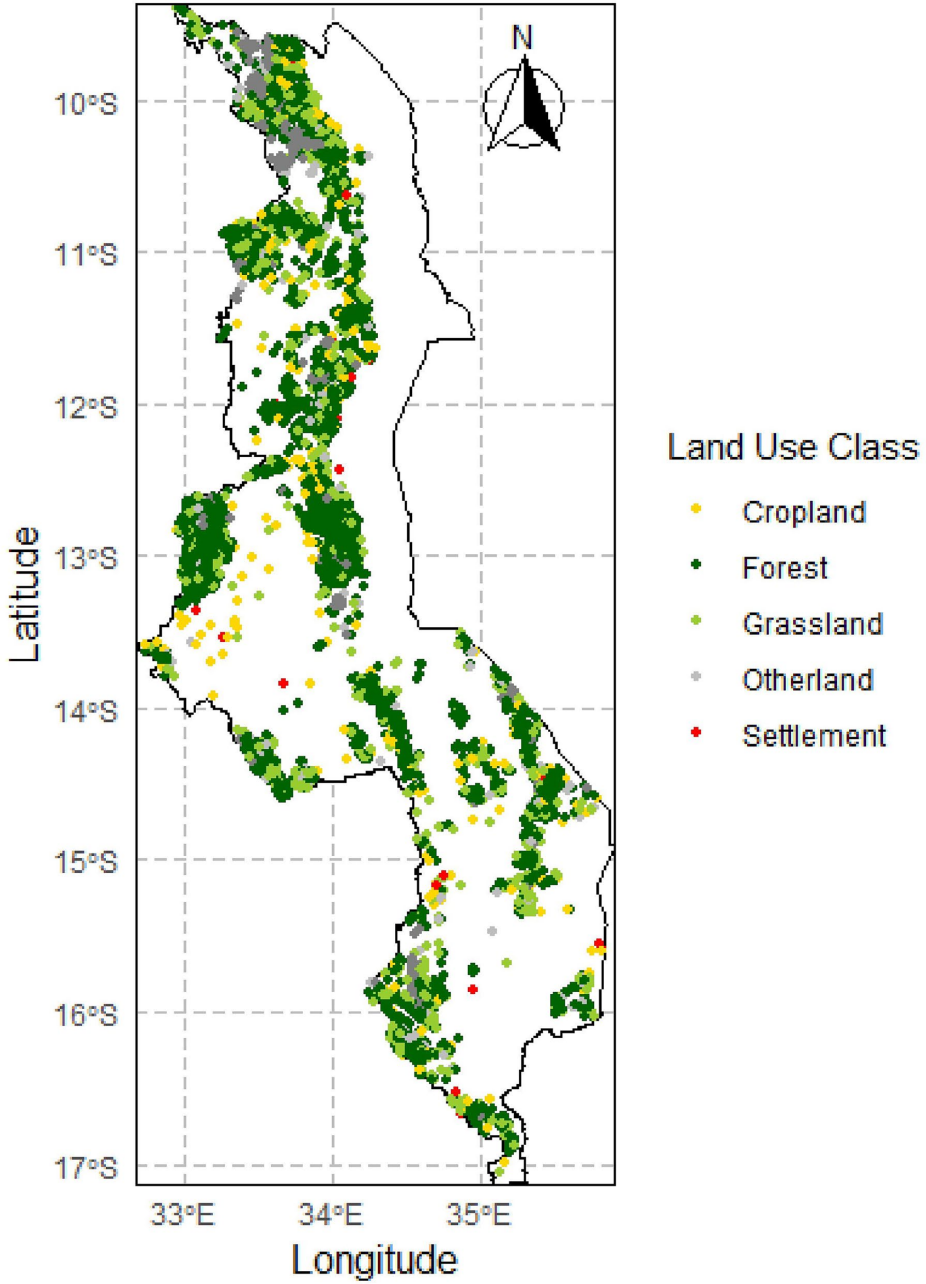
Distribution of NFI monitoring points across Malawi, presented as depicted by the Malawi land use schema for respective land use classes as collected in 2020

The study uses NFI data, which has over 5000 data points across Malawi. The point data was collected in 2010 and 2020 by the Forestry Department from permanent plots. Points for monitoring were selected using a cluster plot sampling approach in the Malawi National Forest Inventory (NFI) campaign (Fig. 2). The primary cluster point was generated within a randomly selected sub-grid, and two additional sampling plots were placed in a ‘T’ shape around the primary point. If the chosen grid sample was inaccessible, primary and alternative replacement points were randomly generated within sub-grids (MCHF, 2021) NFI reference points were independent ground data. Monitoring points were derived by overlaying NFI locations on each global dataset to extract predicted class values. Thus, monitoring points were not a subset of the NFI dataset but comparative samples enabling accuracy assessment.

#### Classification typology between the LULC data products classes and the Malawi land use schema

Correspondence between global dataset class labels (Esri, ESA WorldCover, Dynamic World) and the Malawi NFI schema was harmonised into four consolidated categories (trees, rangeland, crops, built area). Other classes (e.g. water, bare ground, flooded vegetation) were excluded from evaluation or collapsed into rangeland for comparability (Table 1). Classes not directly comparable to NFI (e.g. water, bare ground, flooded vegetation) were excluded from accuracy evaluation or consolidated into rangeland solely for agreement diagnostics; this choice is documented in the code archive to ensure reproducibility.

**Table 1:** Classification typology between the LULC data products classes and the Malawi land use schema from the NFI data included in this study

LULC class (ESRI, ESA and DW)	Class description (standardised from the three data products)	Malawi land use schema
Crops	Humans planted/plotted cereals, grasses, and crops not at tree height; examples: corn, wheat, soy, and fallow plots of structured land	Cropland
Trees	Any significant clustering of tall (~ 15 feet or higher) dense vegetation, typically with a closed or dense canopy; examples: wooded vegetation, clusters of dense, tall vegetation within savannas, plantations, swamp or mangroves (dense/tall vegetation with ephemeral water or canopy too thick to detect water underneath)	Forest
Rangeland	Open areas covered in homogeneous grasses with little to no taller vegetation; wild cereals and grasses with no apparent human plotting (i.e. not a plotted field); examples: natural meadows and fields with sparse to no tree cover, open Savanna with few to no trees, parks/golf courses/lawns, pastures. The mix of small clusters of plants or single plants dispersed on a landscape that shows exposed soil or rock; scrub-filled clearings within dense forests that are not taller than trees; examples: moderate to sparse cover of bushes, shrubs and tufts of grass, savannas with very sparse grasses, trees or other plants	Grassland/shrubland
Built area	Human-made structures, major road and rail networks, and large homogeneous impervious surfaces, including parking structures, office buildings, and residential housing, such as houses, dense villages/towns/cities, and paved roads and asphalt	Settlement

**Fig. 3:**
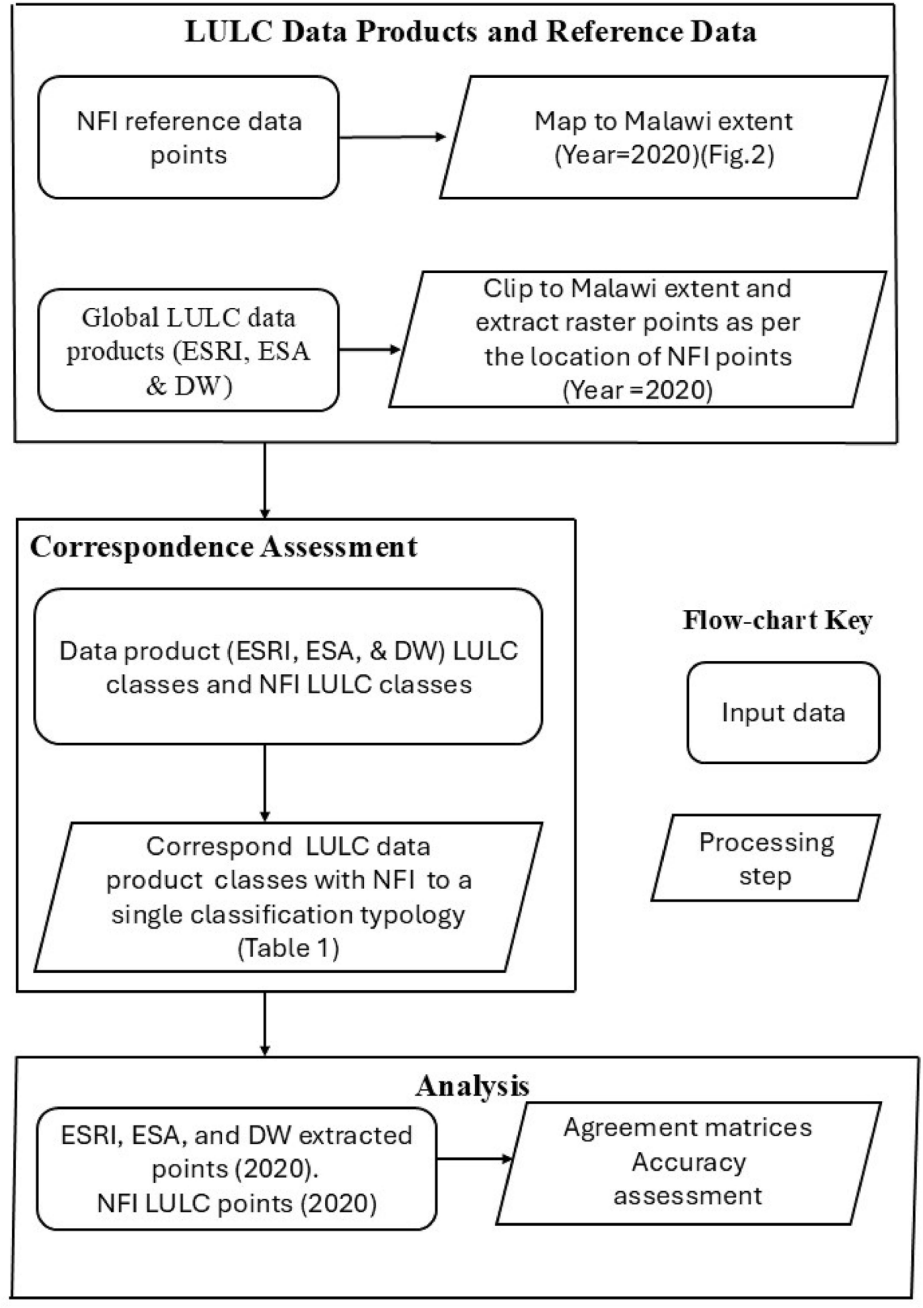
Workflow of methods used in this study. Data from three global LULC products (Esri Land Cover, ESA WorldCover, and Dynamic World) were harmonised with Malawi’s National Forest Inventory (NFI). Steps included class correspondence assessment, extraction of monitoring points, generation of agreement matrices, and accuracy assessment (OA, UA, PA)

The overall method and materials used in this study to evaluate the effectiveness of the datasets relative to the reference NFI data for Malawi, including the analysis of results, are presented in the flowchart (Fig. 3).

#### Accuracy assessment

Agreement between global datasets and NFI reference data was evaluated using multiclass confusion matrices for the four consolidated categories (trees, rangeland, crops, built area). For each product, confusion matrices were cross-tabulated with the predicted classes against the NFI observations. Three accuracy metrics were reported in accordance with best practice (Olofsson et al., 2014).

Overall accuracy (OA) is the proportion of correctly classified samples across all classes. User accuracy (UA) is the proportion of correctly classified samples within each mapped class (precision). Producer’s accuracy (PA) is the proportion of reference samples correctly identified within each class (recall).

Our accuracy estimates are based on design-unadjusted proportions with 1000-draw bootstrap confidence intervals. The NFI employed a national cluster design; however, area-adjusted estimators (Olofsson et al., 2014) require probability sample weights at the class level, which were not available. We therefore report design-unadjusted accuracies, highlight this as a limitation, and provide row-proportion matrices to aid interpretation. To test for error asymmetry, Bowker’s symmetry test was applied to the complete matrix, while per-class McNemar tests assessed bias in pairwise misclassifications.

Classes not directly comparable to NFI (e.g. water, bare ground, flooded vegetation) were excluded from the accuracy evaluation. These exclusions were necessary because NFI reference data does not provide equivalent categories. Because forest landscape restoration in Malawi also targets wetland systems, including dambos, the results reported here do not inform the accuracy of the global products for wetland classes. Analyses therefore focus on four harmonised classes: trees, rangeland, crops, and built area.

## Results

### Sample sizes by class

The sample sizes by class for the NFI 2020 reference points and mapped counts, after extraction from the three global products, show that trees and rangeland were the most common classes in both the reference and map data (Table 2). Crops and built areas were less represented. These imbalances in sample sizes are important for interpreting accuracy metrics, as they affect the accuracy of class-level users and producers.

**Table 2:** Sample sizes by land cover class for the 2020 National Forest Inventory (NFI) reference validation sample points and mapped class points after extraction for Esri Land Cover (ESRI), ESA WorldCover (ESA), and Dynamic World (DW). Row and column totals are shown

Class	NFI Points	ESRI mapped points	ESA mapped points	DW mapped points	Row total
Trees	3521	2790	2358	1548	10,217
Rangeland	889	1863	2132	2942	7826
Crops	273	23	176	165	637
Built area	32	15	4	25	76
Column total	4715	4691	4670	4680	18,756

#### Agreement matrices

**Fig. 4:**
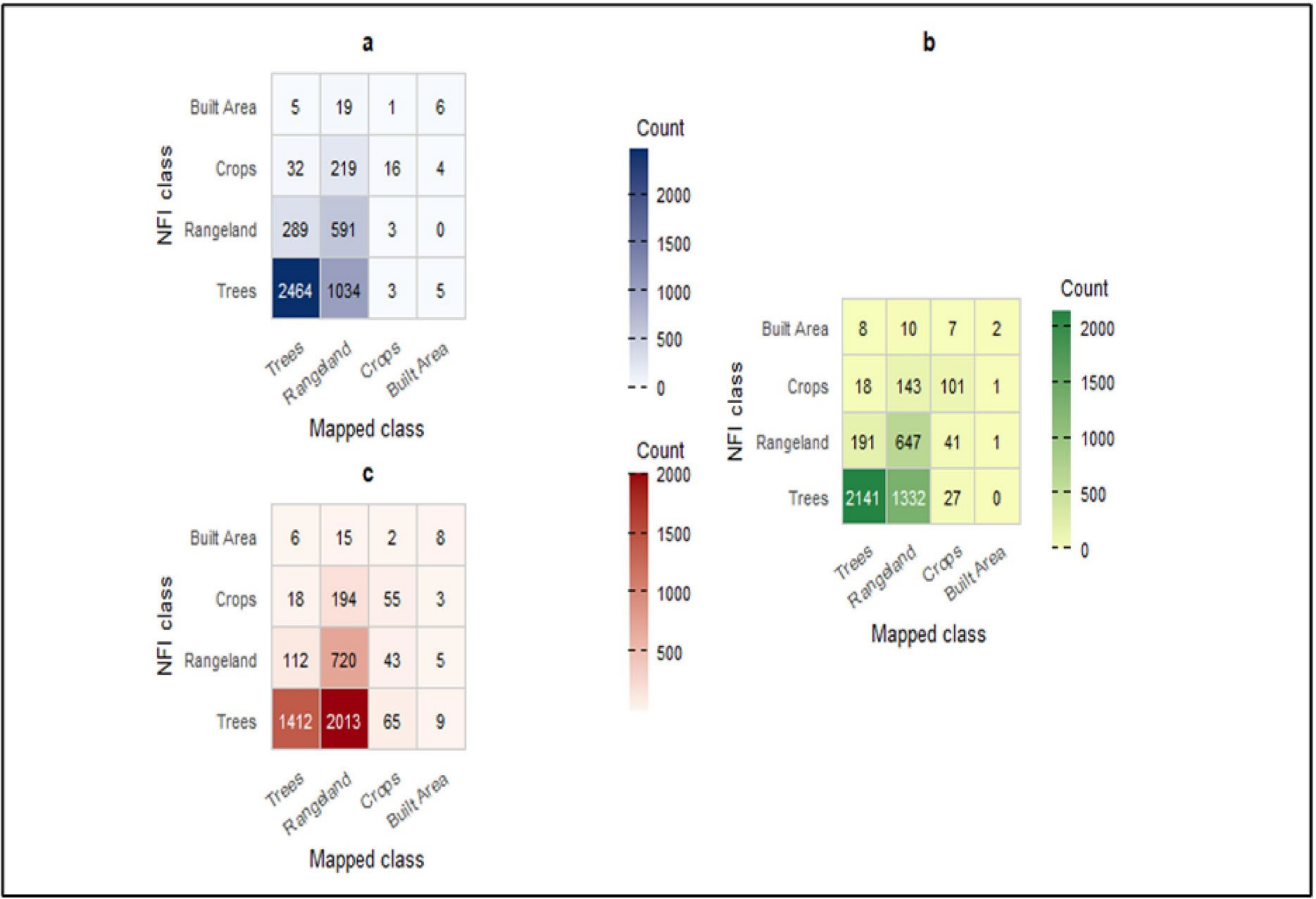
Agreement matrices comparing mapped classes from **a** Esri Land Cover, **b** ESA WorldCover, and **c** Dynamic World against Malawi’s National Forest Inventory (NFI). Colours denote cell frequencies (darker, higher agreement). Rows, NFI classes; columns, mapped classes. Classes not directly comparable to NFI (water, bare ground, flooded vegetation) were excluded; see ‘Materials and methods’

The agreement matrices for Esri Land Cover (ESRI), ESA WorldCover (ESA), and Dynamic World (DW) are each shown (Fig. 4). Each panel represents (a) Esri counts, (b) ESA counts, and (c) DW counts with totals by row and column against NFI counts. These visualisations provide a detailed view of how mapped classes correspond to NFI reference data across the four harmonised land cover categories (trees, rangeland, crops, built area). In all three products, trees and rangeland dominate the samples, while cropland and built-up classes are less represented.

For Esri points (Fig. 4a), the dominant errors involved NFI cropland and built-up points being misclassified as rangeland or trees. ESA (Fig. 4b) showed a similar pattern, but with a stronger tendency to conflate tree cover with rangeland, leading to an underestimation of dense forest cover. DW (Fig. 4c) was particularly prone to over-allocation of cropland and built-up classes into rangeland, while still capturing tree cover with reasonable fidelity.

### Misclassification flows and spatial correspondence

**Fig. 5:**
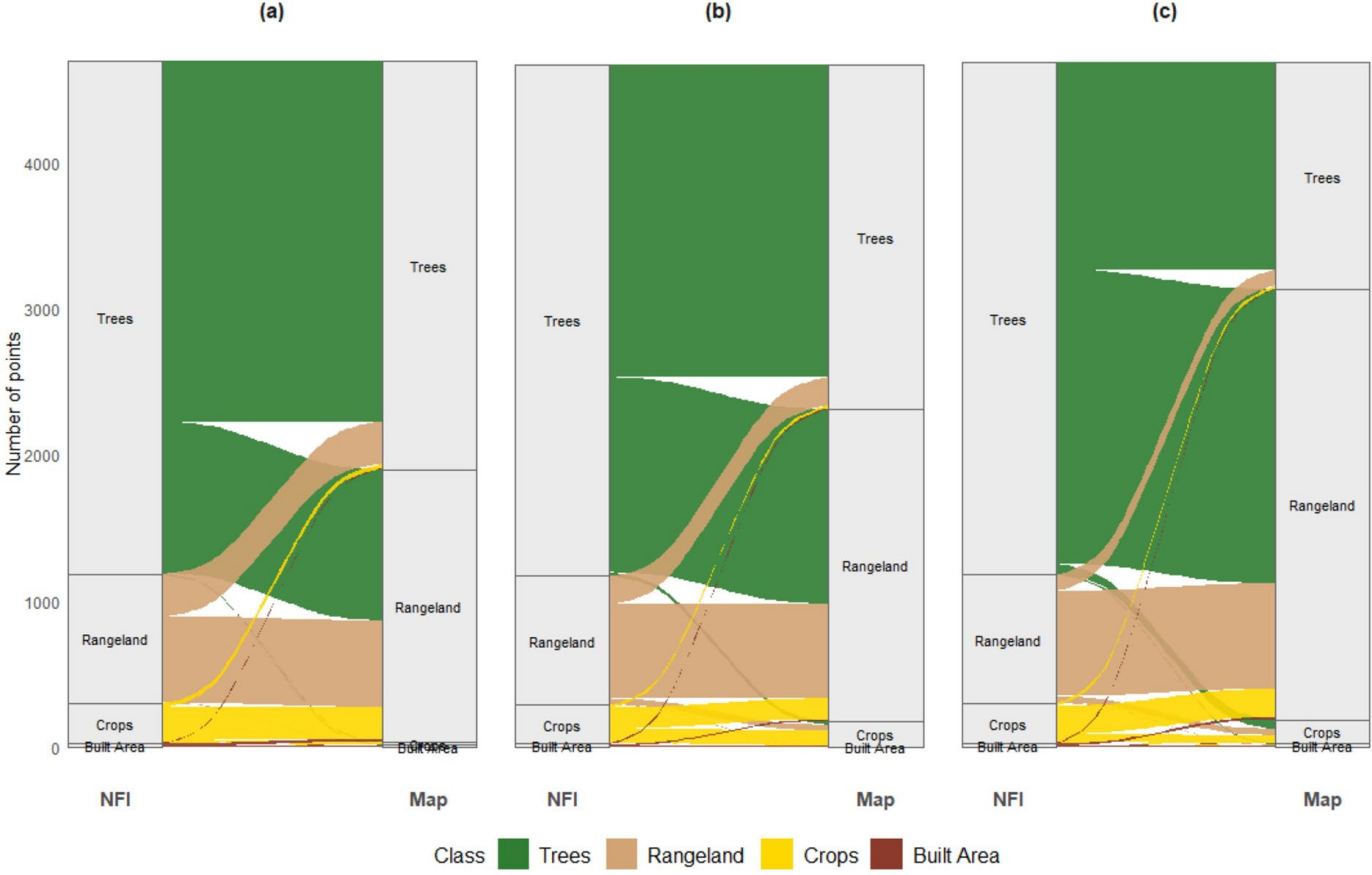
Transition flows from NFI reference classes (left) to mapped classes (right) for **a** Esri Land Cover, **b** ESA WorldCover, and **c** Dynamic World, for Malawi in 2020. Bandwidths are proportional to the number of points. Colours denote NFI classes: trees (green), crops (yellow), rangeland (light gold), built area (brown-reddish)

The flow diagrams illustrate how NFI reference classes are redistributed into mapped classes by each global data product (Fig. 5). Across all three products, the dominant structural pattern is a systematic redistribution of points towards the rangeland class, accompanied by under-detection of crops and built area.

Esri Land Cover (Fig. 5a) flow shows relatively strong retention of the NFI trees class, with the majority of NFI tree points remaining mapped as trees. However, a substantial proportion of NFI rangeland points transition into the trees class, indicating over-allocation of tree cover at the expense of rangeland. This pattern is more pronounced in Esri than in the other two products. Misclassification of NFI crops and built area is also evident. A large share of cropland points transitions into rangeland, with a smaller but visible flow into trees. Built area points are poorly retained, with most flowing into crops, rangeland, or trees rather than remaining in the built area class. This indicates that Esri Land Cover captures dominant woody cover relatively well but struggles to differentiate between open agricultural, rangeland, and settlement classes. Esri Land Covers’ primary misclassification mechanism is tree–rangeland confusion, combined with systematic absorption of minor classes into broader vegetated categories.

The ESA flows (Fig. 5b) display a similar, but more balanced, redistribution pattern. Retention of the NFI trees class remains strong, but transitions from trees into rangeland are more pronounced than in Esri. Conversely, fewer NFI rangeland points transition into trees, suggesting less aggressive over-mapping of tree cover relative to Esri. Crops are again predominantly redistributed to rangeland, though a slightly larger fraction is retained as crops than in Esri. Built area points show very limited retention and are largely absorbed into crops and rangeland, indicating persistent difficulty in detecting settlements at the point scale. Compared to Esri, ESA shows greater bidirectional exchange between trees and rangeland, rather than a dominant one-way flow. This suggests that WorldCover is more conservative in assigning tree cover but still struggles with class separation in mosaic landscapes.

DW (Fig. 5c) exhibits the most pronounced redistribution away from the NFI reference classes. Flows from NFI trees into rangeland are markedly larger than in either Esri or ESA, indicating substantial under-detection of tree cover. At the same time, large flows from crops and built area into rangeland dominate the diagram. Retention of crops and built area is weakest in DW, with only small fractions remaining in their original classes. The rangeland class effectively functions as a sink, absorbing misclassified points from all other classes. Therefore, DW has the highest level of class collapse, with extensive over-allocation to rangeland and limited fidelity to the NFI reference data.

All in all, rangeland is consistently overpredicted across all three data products. Crops and built area are systematically underdetected, with points redistributed primarily into rangeland and, to a lesser extent, trees. Tree cover is best retained in Esri.

**Fig. 6:**
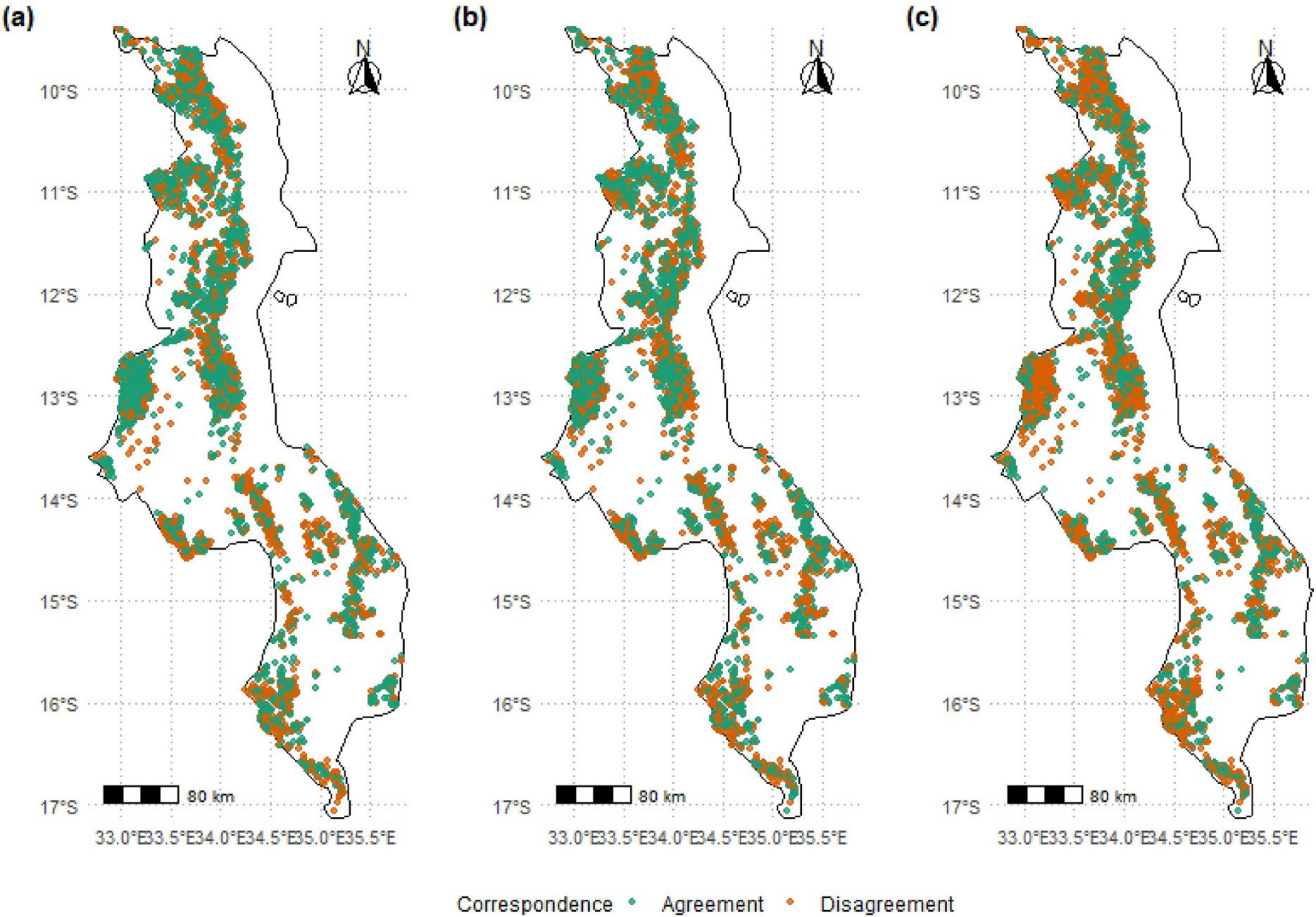
Spatial correspondence between global land cover datasets and Malawi’s National Forest Inventory (NFI) reference points in 2020. Agreement (green) and disagreement (red) are shown for **a** Esri Land Cover, **b** ESA WorldCover, and **c** Dynamic World

The spatial correspondence maps provide a complementary spatial perspective on agreement and disagreement between the global datasets and NFI reference points (Fig. 6). Agreement (green) and disagreement (red) are explicitly mapped, allowing a visual assessment of where classification challenges are concentrated.

Esri Land Cover (Fig. 6a) shows the highest density of agreement points across Malawi. Disagreements are present but appear more spatially dispersed, with fewer contiguous clusters of misclassification. Agreement is particularly strong in areas dominated by tree cover. ESA (Fig. 6b) shows a higher proportion of disagreement points than Esri, with more visible clustering in central and southern Malawi. These clusters correspond to areas characterised by mixed land uses, where cropland, rangeland, and scattered trees coexist. DW (Fig. 6c) exhibits the highest density and spatial concentration of disagreement points. Large contiguous areas of red points are observed across multiple regions, indicating widespread discrepancies relative to the NFI reference data. This spatial signal mirrors the flow-diagram results, confirming that Dynamic World’s extensive reallocation into rangeland yields spatially coherent misclassification rather than isolated errors.

#### Overall accuracy across products

Based on the extracted sample points, accuracy was assessed for each product using overall accuracy (OA), user’s accuracy (UA), and producer’s accuracy (PA) with 1000-draw bootstrap confidence intervals (Table 3). The global land cover datasets demonstrated variable performance when benchmarked against Malawi’s NFI reference data. The Esri Land Cover product achieved the highest overall accuracy (OA), with 65.6% of samples correctly classified and a 95% confidence interval of 64.2 to 67.0%. ESA WorldCover performed moderately, with an OA of 61.9% (95% CI 60.5 to 63.3%). In contrast, Dynamic World showed lower agreement with reference data, recording an OA of only 46.9% (95% CI 45.6 to 48.4%).

**Table 3:** Overall accuracy (all classes) and class-specific producer’s and user’s accuracy (per cent) for Esri Land Cover (ESRI), ESA WorldCover (ESA), and Dynamic World (DW), benchmarked against the 2020 National Forest Inventory (NFI) reference points. Values are design-unadjusted, with 1000-draw bootstrap 95% confidence intervals in parentheses

Metric	ESRI	ESA	DW
Overall accuracy (all classes)	65.6 (64.2 to 67.0)	61.9 (60.5 to 63.3)	46.9 (45.6 to 48.4)
Producer’s accuracy (built area)	19.8 (6.5 to 35.7)	7.2 (0.0 to 18.5)	26.1 (11.4 to 42.3)
Producer’s accuracy (crops)	5.9 (3.1 to 8.8)	38.2 (32.6 to 43.7)	21.3 (16.3 to 26.2)
Producer’s accuracy (rangeland)	66.9 (63.6 to 70.0)	73.5 (70.4 to 76.3)	81.6 (79.0 to 84.0)
Producer’s accuracy (trees)	70.3 (68.8 to 71.8)	61.2 (59.5 to 62.8)	40.4 (38.9 to 42.0)
User’s accuracy (built area)	40.3 (14.3 to 68.8)	49.0 (0.0 to 100.0)	32.1 (14.8 to 52.0)
User’s accuracy (crops)	69.5 (48.3 to 87.5)	57.3 (50.3 to 64.8)	33.4 (26.4 to 40.5)
User’s accuracy (rangeland)	31.7 (29.6 to 34.0)	30.4 (28.4 to 32.4)	24.3 (22.9 to 26.0)
User’s accuracy (trees)	88.3 (87.1 to 89.5)	90.8 (89.6 to 91.9)	91.2 (89.8 to 92.6)

Class-specific accuracies revealed systematic asymmetries across all three products (Table 3). All three datasets achieved high user’s accuracy (UA > 88%), indicating that pixels classified as ‘trees’ were usually correct. However, producer’s accuracy (PA) varied: Esri correctly identified 70.3% of NFI forest points, ESA 61.2%, while Dynamic World performed poorly at 40.4%. This suggests that while tree cover predictions were generally reliable, a substantial proportion of actual tree points were omitted, particularly by Dynamic World.

Both Esri and ESA consistently overpredicted rangeland, with low UA values of 31.7% and 30.4%, respectively. Their PA values were higher (66.9% and 73.5%), showing that most NFI rangeland points were captured, but many other classes were incorrectly mapped as rangeland. Dynamic World exhibited the same trend, with a very high PA (81.6%) but an extremely low UA (24.3%), reflecting pervasive over-allocation of pixels to the rangeland class.

Crops were consistently underestimated across all products. Esri achieved only 5.9% PA for cropland, ESA improved to 38.2%, while Dynamic World remained low at 21.3%. UA values varied, ranging from 33.4 to 69.5%, confirming that cropland was both omitted and misclassified as surrounding vegetation.

Built areas were systematically under-detected. Esri reached 19.8% PA, ESA just 7.2%, and dynamic world 26.1%. UA values were unstable due to the small number of built-up samples, resulting in wide confidence intervals (e.g. ESA UA = 49.0% [0 to 100%]).

Where a class was sparsely sampled, particularly built area, the user accuracy confidence intervals are very wide, and the corresponding estimates should be treated as statistically unreliable. These results demonstrate that while all three datasets perform reasonably well for tree cover, they systematically underrepresent cropland and built-up areas and overgeneralise rangeland. These class-level weaknesses have direct implications for restoration monitoring, where accurate detection of agricultural and settlement land uses is critical for estimating land availability. The error structure is not symmetric. Bowker’s test of marginal symmetry rejected the null of equal off-diagonal misclassification for all three products (*p* < 0.001), indicating that misclassifications are not exchanged in balanced pairs but flow predominantly in one direction. Per-class McNemar tests confirmed that this directional bias operates against crops and built area: for all three products, the probability that crops or built area are mapped as rangeland is significantly greater than the reverse (*p* < 0.001), and for Esri and ESA the same one-way bias is observed against trees being mapped as rangeland. In other words, rangeland functions as a structural sink in the global products, absorbing classes that the reference data record as crops, built area, or (for Esri and ESA) trees, with no statistically detectable flow in the opposite direction.

## Discussion

### Effectiveness of LULC datasets for monitoring FLR targets in Malawi

There is an urgent call for input towards a more measurable definition of integrity and methods to track goals and indicators through 2050 under the post-2020 Global Biodiversity Framework (GBF) (Hansen et al., 2021). For countries such as Malawi, where data gaps and institutional capacity constraints are significant, this call is especially urgent. The GBF explicitly requires monitoring systems that can track ecosystem extent, integrity, and restoration progress with spatially consistent and repeatable metrics. Global land use and land cover (LULC) datasets such as Esri Land Cover, ESA WorldCover, and Google’s Dynamic World aim to address these gaps by providing annual, high-resolution (10 m) products at continental and global scales. However, the reliability of these datasets for national forest and landscape restoration (FLR) monitoring has been challenged. Recent reviews have emphasised the importance of validating such products against authoritative national reference data to ensure that reported trends and baselines are credible (Felden et al., 2023; Reis et al., 2019; Wijaya et al., 2019). This study responds to that need by evaluating three global products against Malawi’s National Forest Inventory (NFI), the most authoritative national dataset currently available.

None of the three products reached accuracy levels sufficient for robust FLR monitoring in Malawi’s heterogeneous mosaics (Table 3). The values are consistent with recent comparative studies showing that global 10-m datasets tend to underperform in Africa’s smallholder-dominated landscapes (Kerner et al., 2024; Xu et al., 2024) and are substantially lower than the accuracies reported by product developers (Esri reports global accuracies above 85%; Karra et al., 2021). Performance at national scales, especially in complex agricultural mosaics, can therefore diverge substantially from global averages.

Because these accuracies are design-unadjusted, they cannot be assumed to be representative of the country. For national reporting, this is a material caveat, since headline accuracy figures produced without design-based inference may not generalise beyond the sampled points. The dominance of tree and rangeland classes in both the reference and mapped data (Table 2) shaped the error structure, with cropland and built-up classes consistently absorbed into the larger categories (Figs. 4 and 5). Errors were not random but spatially structured, concentrated in central and southern Malawi where smallholder mosaics dominate (Fig. 6).

Each dataset showed distinct misclassification patterns. Esri Land Cover, although the most accurate overall, systematically underestimated built-up areas and cropland, which led to an overestimation of rangeland extent. ESA WorldCover exhibited greater confusion between tree cover and shrubland or grassland, leading to an underestimation of dense forest patches. Dynamic World performed the worst, with widespread overestimation of cropland and built-up areas, frequent misclassification of rangeland, and particularly low accuracy in smallholder landscapes. These differences stem from their underlying methodological approaches. For example, Dynamic World was designed to provide near-real-time classifications based on Sentinel-2 imagery and machine-learning probabilities. While this is useful for global change detection, its probabilistic classification system appears ill-suited for capturing the subtle land use gradients typical of Malawi’s mosaics. Conversely, ESA WorldCover employs a different algorithmic pipeline and validation strategy, which may explain its marginally better performance with tree cover but weaker ability to represent agricultural diversity.

These findings align with a growing body of evidence indicating that heterogeneous landscapes with high anthropogenic influence pose significant challenges for global mapping (Kerner et al., 2024). In Malawi, rangelands are not homogeneous: they include communal grazing lands, fallows, degraded woodlands, and shrublands. Their spectral signatures often overlap with open woodland and fallow cropland, creating systematic confusion between ‘Trees’ and ‘Rangeland’. Similarly, built-up areas in rural Malawi are typically small, scattered homesteads with high spectral mixing of roofs, bare soil, and vegetation, making them difficult to capture at 10-m resolution. These structural realities explain why the datasets systematically under-detected cropland and built-up classes, while inflating rangeland.

While these misclassifications are problematic, it is important to acknowledge the strengths of global products. They offer full spatial coverage, annual consistency, and temporal depth that are otherwise unattainable in Malawi due to financial and institutional constraints. They also provide a valuable baseline for initial assessments of land cover change. For example, Esri and ESA products both captured broad spatial patterns of tree cover in the north and central regions of Malawi. Dynamic World’s near-real-time classification could be helpful for rapid deforestation alerts, even if its class-level accuracy is low. Thus, the issue is not whether global products are helpful, but rather how they should be used.

Our findings support the idea that global datasets are valuable tools for national FLR monitoring, but only if their limitations are clearly understood and addressed. The systematic misclassifications we observed have direct consequences for policy and reporting. Cropland and built-up areas, which are consistently misclassified as rangeland, are the same land uses where restoration efforts are often targeted such as increasing tree cover on croplands or establishing agroforestry along farm boundaries. If these areas are underdetected, national restoration strategies risk overestimating the land available for interventions and underestimating progress already made. Likewise, confusion between rangeland and tree cover could lead to misleading figures of tree cover gains or losses. These limitations underscore the need for Malawi to adopt integrated monitoring strategies that leverage global dataset coverage while grounding them in the reliability of NFIs and the contextual accuracy of local data. In operational terms, such a framework would use the global products for wall-to-wall screening and change flagging, retain the NFI plots as the design-based reference for validation and area estimation, and draw on district-level records such as agroforestry adoption, woodlot inventories, and traditional authority land records to verify the crops and built area classes that the global products miss. This balanced approach is necessary if Malawi is to meet the urgent demand for credible indicators under the GBF and track its progress towards ambitious FLR targets through 2050.

### Implications for FLR monitoring

These accuracy patterns carry four direct implications for Malawi’s ability to monitor and report on FLR progress (Grantham et al., 2020; Xu et al., 2024). Because a single-date classification forms the basis for any later change or trend derived from the same product, robust accuracy assessment is a prerequisite for credible area and change estimation (Olofsson et al., 2014).

First, misclassification undermines the credibility of national reporting under international frameworks such as the GBF, REDD+, and NDCs. Overestimating rangeland loss or underestimating cropland tree cover could distort baseline conditions and inflate restoration opportunities, a concern already noted in restoration reporting debates (Felden et al., 2023; Hansen et al., 2021). Under-reporting tree cover on farmland may undervalue agroforestry contributions to FLR targets (Djenontin et al., 2022; Hermans et al., 2020). Conversely, exaggerated estimates of tree cover gains could falsely suggest progress where little has occurred, as highlighted by comparative assessments of global LULC datasets (Kerner et al., 2024; Xu et al., 2024).

Second, misclassifications hinder resource allocation. FLR interventions are often prioritised based on degradation severity and land use type (Dey & Schweitzer, 2014; Mansourian & Stephenson, 2023). If areas classified as rangeland in global products are, in fact, cropland or mixed agroforestry, interventions might be misdirected, reducing their effectiveness. For example, efforts to expand community forests or reforest degraded lands could be aimed at areas already covered with trees, while neglecting degraded cropland in urgent need of attention (Guariguata & Evans, 2020).

Third, the differences across products highlight the risk of dataset shopping, where decision-makers select the dataset that best fits their narrative. With Esri indicating tree gains, ESA highlighting forest underestimation, and Dynamic World exaggerating rangeland, conflicting evidence can be used selectively to justify specific policies. Transparent validation against NFIs is critical to prevent this (Olofsson et al., 2013, 2014).

Fourth, while GLAD loss/gain products provide valuable insights into deforestation events, they do not sufficiently capture the complex dynamics of FLR in Malawi’s mosaics. Restoration involves not only forest gain but also increasing tree cover in agricultural and settlement landscapes, which GLAD is not designed to measure (Potapov et al., 2022; Tulbure et al., 2022). This underscores the necessity of adapting global datasets to local definitions of success (Sun et al., 2022).

Returning to the cited estimate that Malawi loses around 33,000 ha of forest annually (Skole et al., 2021), our findings suggest that such national figures are likely to be directionally useful but numerically uncertain. Moderate accuracy and systematic confusion among trees, rangeland, and cropland indicate that single-source estimates of forest loss in mosaic landscapes should be treated with caution and cross-checked against multiple data streams before being incorporated into FLR planning and reporting.

Lastly, these findings demonstrate that global datasets are not wrong, but incomplete. They offer valuable coverage and temporal consistency, but their class definitions, algorithms, and training data are not tailored to Malawi. Their optimal use is to complement, not replace, national systems. Combining them with NFI benchmarks, community-based observations, and remote sensing innovations can establish a pluralistic monitoring system that fulfils both scientific standards and policy requirements (Fagan et al., 2020; Reis et al., 2019; Wijaya et al., 2019).

## Limitations and future directions

Three limitations should be acknowledged when interpreting the results presented above. First, area-adjusted accuracy estimators (Olofsson et al., 2014) could not be applied because the NFI cluster design’s class-level probability weights were not made available to us; we therefore report design-unadjusted accuracies with bootstrap confidence intervals and explicitly flag this as a constraint on the inferential reach. Second, accuracy assessment at 10-m pixel resolution against point-based reference data is potentially sensitive to positional misregistration: an NFI point recorded on the boundary of two adjacent land covers can be allocated to either pixel depending on the geolocation precision of the underlying Sentinel-2 imagery (Gascon et al., 2017), particularly in highly fragmented landscapes. Third, the harmonisation step required by the differing class schemas across the three global products excluded certain NFI categories (notably Wetlands, including Dambos and other temporarily flooded areas) from the accuracy assessment. These exclusions were unavoidable for inter-product comparability but mean that the present results cannot be used to evaluate global product performance against Malawi’s wetland classes, even though these classes are ecologically and hydrologically important.

Another consideration is that the NFI reference data are treated here as ground truth; yet, like all reference datasets, they may contain classification errors, so the reported values reflect agreement with the NFI rather than with an error-free baseline. The Built Area class relies on only 32 reference points, making its class-level estimates unstable and sensitive to individual points. Three directions are proposed for future work. The first is for subsequent studies to obtain the NFI cluster design weights, enabling area-adjusted accuracy estimation following the Olofsson et al. (2014) protocol and yielding class-level confidence intervals that are properly representative at the national scale. The second is an integration study that combines global remote-sensing products with community-based monitoring observations from district-level FLR actors (for example, agroforestry adoption records, woodlot inventories, and traditional authority records) to test whether locally collected indicators can correct the systematic omissions of crops, built area, and wetlands documented here. The third is a focused calibration study using high-resolution commercial imagery or unmanned aerial vehicle (UAV) flights over a stratified subsample of NFI sites to quantify the contributions of positional misregistration, fragmentation, and spectral mixing to the class-specific biases reported in this analysis. Each direction would strengthen the inferential basis for using global products in national FLR monitoring without requiring a complete redesign of the existing reference architecture.

## Conclusions

The study shows that although they moderately perform well, none of the three global land cover products tested, Esri Land Cover, ESA WorldCover, and Dynamic World, achieve accuracy levels sufficient to monitor FLR in Malawi independently. Esri performed best but still systematically misclassified crops and built area as rangeland or trees. ESA confused tree cover with rangeland, while Dynamic World consistently overestimated rangeland at the expense of other classes. Class imbalances contributed to these misclassifications in both the reference and mapped samples and were spatially patterned across the landscape. Trend analysis also revealed differences between Esri’s annual series and NFI benchmarks, highlighting the risks of relying solely on global datasets to interpret changes in tree cover or rangeland. GLAD loss/gain products may provide additional insights but cannot capture the agroforestry mosaics central to Malawi’s FLR objectives.

Global datasets are useful but incomplete. They offer coverage and consistency but lack the detail and contextual accuracy needed for national FLR planning and reporting. Malawi and similar countries should adopt integrated monitoring approaches that combine global products with NFI data and local knowledge. As an immediate step, the Forestry Department should commission a national validation study using design-based inference with the NFI cluster weights, so that accuracy and area can be reported with nationally representative confidence intervals. Only by integrating these sources can FLR progress be measured reliably, resources allocated effectively, and international commitments fulfilled with confidence.

## Data Availability

The global land-cover datasets analysed in this study are publicly available: Esri 10 m Annual Land Use/Land Cover (via the ArcGIS Living Atlas of the World), ESA WorldCover 10 m (via the ESA WorldCover data portal), and Dynamic World 10 m (available through Google Earth Engine). The reference dataset used for validation (Malawi National Forest Inventory data) was obtained from the Forest Monitoring Unit, Department of Forestry, Government of Malawi, and is not publicly available. Access to the National Forest Inventory data can be requested from the Forest Monitoring Unit (Department of Forestry, Malawi) and may be subject to institutional approval and data-use conditions. Derived data supporting the findings (e.g., harmonised class crosswalks and extracted map values at reference points) and analysis code are available from the corresponding author upon reasonable request.
